# mSphere of Influence: Apoptotic Mimicry and Virus Entry

**DOI:** 10.1128/mSphere.00034-21

**Published:** 2021-01-27

**Authors:** Melinda A. Brindley

**Affiliations:** a Department of Infectious Diseases, Department of Population Health, College of Veterinary Medicine, University of Georgia, Athens, Georgia, USA

**Keywords:** apoptotic mimicry, phosphatidylserine, viral entry

## Abstract

Melinda A. Brindley works in the field of virology with specific interests in understanding how viruses enter cells. In this mSphere of Influence article, she reflects on how the paper “Vaccinia virus uses macropinocytosis and apoptotic mimicry to enter host cells” by J. Mercer and A. Helenius (Science 320:531–535, 2008, https://doi.org/10.1126/science.1155164) made an impact on her by expanding our understanding of virus-host interactions and virus-cell binding.

## COMMENTARY

Early in my career, I was following up on a study that suggested the Tyro3, Axl, Mer (TAM) family members facilitated Ebola virus entry into cells (1). While I could recapitulate the experiments showing production of Axl in cells enhanced Ebola virus glycoprotein (GP)-mediated entry, I was not able to show a biochemical interaction between the Ebola virus glycoprotein and Axl. At that time, dogma taught that all enveloped viruses have viral glycoproteins studded in the lipid membrane which interact with specific cellular proteins or sugars in a lock-and-key-like manner. I tried to detect a biochemical interaction between Ebola virus GP and Axl numerous ways with no success. Then in 2008, Mercer and Helenius published “Vaccinia virus uses macropinocytosis and apoptotic mimicry to enter host cells” (2). Rather than the virus interacting with the cell using a virally encoded protein, the lipids within the viral envelope mediated cellular interaction in a manner similar to apoptotic debris. Apoptotic cells are, in part, recognized by the presence of phosphatidylserine in the outer leaflet of their plasma membrane. Phosphatidylserine receptors on phagocytes bind to the lipid and facilitate engulfment to clear the debris. Vaccinia virus usurps this established cellular pathway to “trick” cells to engulf the virus. Once within internal vesicles, viral fusion proteins are activated, delivering the virus genome to the cytoplasm where virus replication takes place. While the interaction between phosphatidylserine and its receptors is specific, the interaction is not unique to any virally encoded component, and therefore, the mechanism of apoptotic mimicry could be used by any enveloped virus containing phosphatidylserine in the outer leaflet. Since Axl can bind to phosphatidylserine through an adapter protein, Ebola virus, similar to vaccinia virus, can be internalized into cells using apoptotic mimicry. With no lock-and-key-like mechanism involving the Ebola virus glycoprotein, Axl production facilitated Ebola virus internalization through interaction with viral lipids, explaining why my biochemical experiments looking for protein-protein interactions failed (3).

Mercer and Helenius (2) monitored fluorescently tagged vaccinia virus and observed particles were engulfed by cellular blebbing in an actin-dependent process. Further knockdown and inhibitor studies defined the entry process as macropinocytosis, a mechanism used to clean up apoptotic debris. Further studies blocked infectivity by masking virion-associated phosphatidylserine with annexin V. Vaccinia virions could even be delipidated, and then the lipid envelope could be restored with specific lipids. Phosphatidylserine levels were critical to ensure the relipidated particles were infectious, suggesting that vaccinia mature virion (MV) particles interact with the target cell in a manner similar to apoptotic debris and that the phosphatidylserine in the viral membrane mediates binding and internalization.

The first viral apoptotic mimicry papers changed the way scientists think about virus-cell interactions, as they demonstrate that not all viruses require a specific lock-and-key binding event between the viral glycoprotein and cellular receptor for internalization. Subsequent research has shown that while phosphatidylserine-receptor binding may not be a high-affinity interaction, it is sufficient and used by a number of viruses that have wide tropism. As someone who has studied virus entry for my entire career, broadening the definition of a cellular receptor has altered the way we design experiments to monitor virus entry as well as opened up new avenues of investigation. The classic paradigm of viral glycoprotein interacting with a cellular protein at a specific interface enables one to explore and characterize the interface in a pure system, frequently using purified soluble forms of both proteins. Monitoring cellular interactions with viral envelope phospholipids becomes more complicated. Additionally, eukaryotic cells produce a number of proteins that recognize phosphatidylserine, some interacting directly with the lipid and others requiring adapter proteins. Therefore, characterizing virus binding and internalization requires use of intact particles and careful choice of target cells. Additionally, we have started characterizing specific lipids found in the particles and comparing particle lipid composition across several host cell types to further correlate lipid constituents and particle infectivity.

The paradigm shift in virus-host interaction led to identifying a number of viruses that utilize apoptotic mimicry to facilitate entry. Progress was also made in our understanding of cell membrane phosphatidylserine (PS) regulation, which has provided virologists with the means to mechanistically examine how viral envelopes acquire receptor accessible PS. Enveloped viruses acquire their lipid membrane as they bud through a membrane in the infected cell. Phospholipid distribution in cells is highly regulated. The plasma membrane is asymmetric, while sphingomyelin and phosphatidylcholine are predominantly found in the outer leaflet; phosphatidylserine and most of the phosphatidylethanolamine and phosphatidylinositol are sequestered in the inner leaflet. Phospholipid distribution is altered briefly during signaling events or irreversibly during apoptosis. Some proteins involved in lipid transbilayer movement (lipid movement between inner and outer leaflets) were identified in 2013 and 2014. Suzuki et al. identified XK-related protein 8 (Xkr8) as a PS scramblase using a cDNA library screen and isolating cells high in outer leaflet PS (4). Scramblases are proteins that mediated the transbilayer movement of specific lipids in both directions. The following year, Segawa et al. identified PS flippase complexes composed of ATP11C and a chaperone protein, CDC50a (5). Flippases mediate the healthy cell asymmetry by ensuring any PS molecule found in the outer leaflet is flipped to the inner leaflet. With the identification of the cellular players mediating lipid asymmetry, new experiments could be designed to determine whether these cellular proteins alter virion infectivity. Virologists have started characterizing the role of these cellular proteins during virus replication and infectivity.
